# Anticancer Properties and Mechanism of Action of Oblongifolin C, Guttiferone K and Related Polyprenylated Acylphloroglucinols

**DOI:** 10.1007/s13659-021-00320-1

**Published:** 2021-09-29

**Authors:** Christian Bailly, Gérard Vergoten

**Affiliations:** 1 Scientific Consulting Office, OncoWitan, 59290 Lille, Wasquehal France; 2 Inserm, INFINITE - U1286, Faculté de Pharmacie, University of Lille, Institut de Chimie Pharmaceutique Albert Lespagnol (ICPAL), 3 rue du Professeur Laguesse, BP-83, 59006 Lille, France

**Keywords:** Anticancer, Autophagy, HSPA8, Natural products, Polyprenylated acylphloroglucinols

## Abstract

Polyprenylated acylphloroglucinols represent an important class of natural products found in many plants. Among them, the two related products oblongifolin C (Ob-C) and guttiferone K (Gt-K) isolated from *Garcinia* species (notably from edible fruits), have attracted attention due to their marked anticancer properties. The two compounds only differ by the nature of the C-6 side chain, prenyl (Gt-K) or geranyl (Ob-C) on the phloroglucinol core. Their origin, method of extraction and biological properties are presented here, with a focus on the targets and pathways implicated in their anticancer activities. Both compounds markedly reduce cancer cell proliferation in vitro, as well as tumor growth and metastasis in vivo. They are both potent inducer of tumor cell apoptosis, and regulation of autophagy flux is a hallmark of their mode of action. The distinct mechanism leading to autophagosome accumulation in cells and the implicated molecular targets are discussed. The specific role of the chaperone protein HSPA8, known to interact with Ob-C, is addressed. Molecular models of Gt-K and Ob-C bound to HSPA8 provide a structural basis to their common HSPA8-binding recognition capacity. The review shed light on the mechanism of action of these compounds, to encourage their studies and potential development.

## Introduction

Polycyclic polyprenylated acylphloroglucinols (PPAPs) represent an important group of natural products, extremely diversified and present in many plants [1–3]. The PPAPs database include more than 850 structures, with molecules showing extremely diverse bioactivity profiles [4]. There are compounds acting as potent modulators of the major histocompatibility complex, such as PPAPs isolated from *Garcinia bancana* [5], compounds with anti-adipogenic activity, isolated from *Hypericum subsessile* [6], compounds with marked antiparasitic and antimicrobial effects, such as PAPPs identified from the medicinal plant *Symphonia globulifera* [7], and PPAPs with anti-depressive and hepatoprotective activities found in the aerial parts of *Hypericum scabrum* [8], to cite only a few examples. There are also many compounds with anti-inflammatory, anticancer, antifungal, antibacterial, antiviral, immunoregulatory properties and other bioactivities.

There is a botanical family particularly rich in PPAPs: the *Garcinia* plants, with approximately 400 species distributed around the world. A large variety of bioactive molecules have been isolated from the bark, seed, fruits, peels, leaves, and stems of various *Garcinia* species, such as *G. mangostana, G. xanthochymus,* and *G. cambogia* [9]. Several of these species are used in traditional medicine, such as the small tree *G. oblongifolia* Champ. ex Benth (Clusiaceae) which is rich in PAPPs, notably a series called oblongifolins [10–12]. The edible fruits of *G. oblongifolia* contain several oblongifolin derivatives [13]. Natural products isolated from *Garcinia* species can be used to treat multiple diseases and conditions, ranging from obesity to cardiovascular diseases, diabetes, and cancer. The anticancer compounds are particularly abundant and diversified in *Garcinia* species, such as the xanthone gambogic acid [14], mangostin and mangostenol derivatives [15], and mono/biflavonoids [16, 17]. In recent years, several PPAPs have revealed marked anticancer properties and their mechanism of action has been elucidated, at least partially. The present review offers a focus on two promising PPAPs with prominent antitumor effects, oblongifolin C (Ob-C) and guttiferone K (Gt-K). These two structurally related compounds provide novel opportunities to combat cancer and certain chronic diseases.

## Structure and isolation of oblongifolin C and guttiferone K

**Fig. 1:**
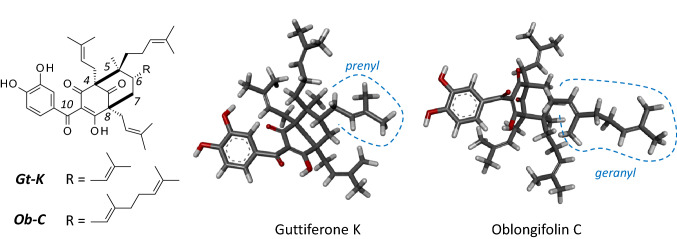
Chemical structures and 3D model of oblongifolin C (Ob-C, C_43_H_58_O_6_, PubChem CID: 102,468,632) and guttiferone K (Gt-K, C_38_H_50_O_6_, PubChem CID: 16,216,670). The two compounds differ by the nature of the C-6 substituent, prenyl (Gt-K) or geranyl (Ob-C), as indicated

Ob-C and Gt-K bear the same 3,4-dihydroxybenzoylphloroglucinol (or bicyclo[3.2.1]octane-2,4,8-trione) core, substituted with a 4-methylpent-3-enyl group at the C5 position and a 3-methylbut-2-enyl group at the C4 and C8 positions. They differ by the nature of the side chain at position C6: Gt-K has a prenyl side chain (C_5_H_9_) whereas Ob-C bears a longer geranyl side chain (C_10_H_17_) (Fig. 1). Consequently, Ob-C is a slightly bigger and more hydrophobic compound than Gt-K. The two molecules are essentially hydrophobic and poorly soluble in water (Table 1).

**Table 1:** Computed physico-chemical properties of Gt-K and Ob-C

Compound	Guttiferone K	Oblongifolin C
Molecular Weight (amu)	602.8	670.9
Dipole moment (D)	4.2	4.6
Total Solvent Accessible Surface Area (SASA) (Å^2^)^a^	901.7	980.1
Hydrophobic SASA	681.4	729.9
Hydrophilic SASA	133.0	148.6
Molecular Volume (Å^3^)	1881.5	2068.0
Donor Hydrogen Bonds	3	3
Acceptor Hydrogen Bonds	6	6
log P (octanol/water)	7.1	8.2
log S (aqueous solubility)	−7.6	−8.5

Drug properties were calculated with the BOSS 4.9 software. The indicated values are for the C-10 keto tautomeric form for the two molecules

^a^SASA calculated with a probe of 1.4 Å radius

**Fig. 2:**
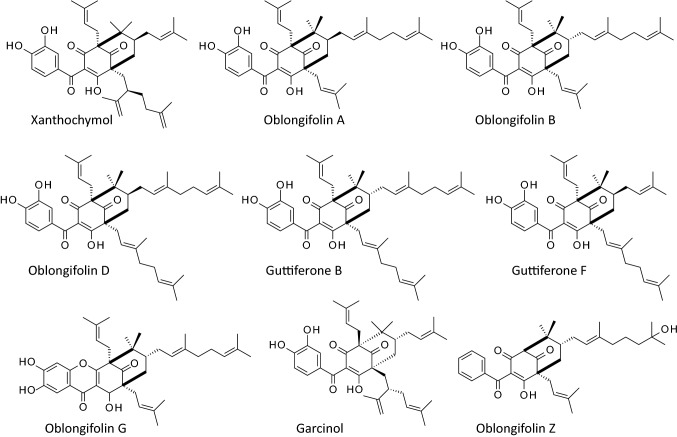
Structures of selected compounds

There are 21 guttiferone derivatives (designated Gt-A to Gt-U) and many variants, such as oxy-Gt-K or K2, oxy-Gt-A, -I, -M, 2,16-oxy-Gt-A [18] and many others such as Gt-BL [19]. Similarly, there are about 30 oblongifolin derivatives (named Ob-A to Ob-Z, and Ob-AA and variants) inventoried in the PPAPs database. The structures of selected compounds are presented in Fig. 2. Oblongifolins have been mainly isolated from plants of the *Garcinia* family (Guttiferae). The first compounds Ob-A-to-D were isolated in 2006 from the bark of *G. oblongifolia* collected in Vietnam, hence the name oblongifolin [20]. Ob-E-to J were isolated from the same plant a few years later, together with structurally related compounds such as oblongixanthones A-C, garcicowin B and nigrolineaxanthone T [10, 21]. Oblongifolins can be found in the bark but also in the leaves of *G. oblongifolia*, used also to isolate Ob-J-to-U [22]. Occasionally, oblongifolins have been found in other species of *Garcinia*, such as Ob-C discovered in *G. yunnanensis* together with the xanthone derivatives garciyunnanins A and B [23]. This bioactive compound Ob-C, the most studied in the oblongifolin series, has been also isolated from the stem of *G. schomburgkiana* Pierre together with the related compound schomburgbiphenyls A and B [24].

Guttiferones can be found in a broad variety of plants. For examples, Gt-A and Gt-G have been found in the twigs of *Garcinia macrophylla* [25]. Gt-H was found in the fruits of *Garcinia xanthochymus* [26] and Gt-E from the fruits of *Garcinia pyrifera* [27]. Gt-I can be isolated from the bark and stem of *Garcinia humilis* [28] and from the stem bark of *Garcinia griffithii* [29]. The compounds can also be found in other plant species. Gt-A was found in the seed shells of *Symphonia globulifera* [30] and Gt-F in the stem bark of *Allanblackia gabonensis* [31]. Here we will essentially focus on the potent compound Gt-K which has been isolated from the fruits of *Garcinia cambogia* [32] and *Garcinia yunnanensis* [33], from the fruits of *Rheedia calcicola* [34], from the stem bark of *Rheedia acuminata* [35] and a few other plants.

Some plant materials contain both types of compounds. This is the case of the Brazilian red propolis which was found to contain Gt-E and Ob-A [36, 37]. Similarly, Gt-E and Ob-B have been found recently in *Symphonia globulifera* L.f. (Clusiaceae) [38]. These compounds are natural products, usually obtained by plant extraction methods. But the total synthesis has been reported as well, offering chemical alternatives to obtain large quantities of the compounds and providing opportunities to produce structural analogues and derivatives [3, 39]. For examples, structural variations of the benzophenone moiety of Ob-C afforded a series of derivatives with a reinforced activity against the c-Met enzyme [40]. Nevertheless, these are complexes, multi-steps syntheses. The overall yield for the total synthesis of Ob-A is about 6%, but it represents a convenient approach to define precise structure–activity relationships [41]. Similarly, the stereoselective synthesis of Gt-A is feasible, but it is a long (13 steps) and difficult procedure [42]. Hemisynthetic derivatives have been prepared as well [43, 44].

**Fig. 3:**
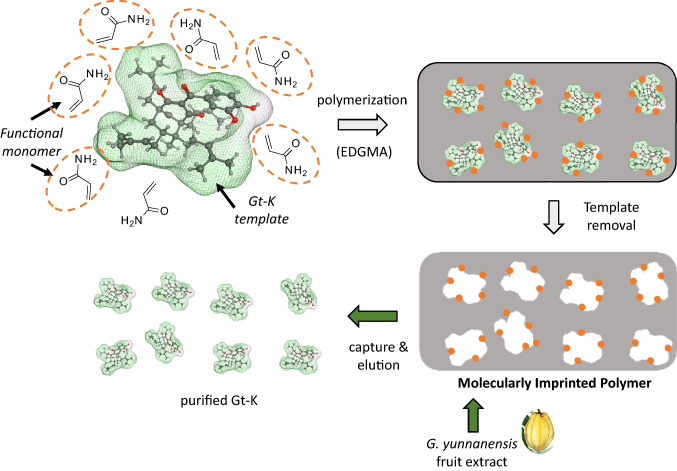
The molecularly imprinted solid-phase extraction (MISPE) procedure used to purify Gt-K from a fruit extract of *G. yunnanensis*. The molecularly imprinted polymers (MIP) is prepared using acrylamide as a functional monomer and ethylene glycol dimethacrylate (EDGMA) as a crosslinking agent, to obtain the Gt-K-specific template. The template is then used to purify the natural products by solid phase chromatography upon elution with different methanol/water mixtures. Unwanted molecules are eluted first (with MeOH/H_2_O: 35/65) followed by Gt-K (50/50) and Ob-C (70/30) (more details in [45])

The most efficient approach to produce appreciable quantities of both Ob-C and Gt-K consists of using molecularly imprinted polymers (MIPs) to obtain the two products successively from fruit extracts of *Garcinia yunnanensis* Hu. These MIPS are porous materials, prepared from a bulk polymerization with a functional monomer (here acrylamide) and a crosslinking agent (ethylene glycol dimethacrylate), which can be employed as adsorbents for the solid phase extraction of natural products, as illustrated in Fig. 3. In the present case, 5 g of *G. yunnanensis* fruit extracts and 2 g of MIPs led to the isolation of 140 mg of Ob-C and 46 mg of Gt-K with a purity of 95% and 88% respectively [45]. This is a very convenient and clean method (extraction with a water/methanol gradient) to obtain the desired compounds. MIPs are particularly well adapted to purify secondary metabolites from plants [46].

It is important to mention that the structural elucidation with these PAPP compounds is not always easy due to the existence of various isomeric forms. For example, the compounds Gt-E and xanthochymol (Fig. 2) both isolated from *G. xanthochymus* are two π-bond benzophenone isomers which are difficult to separate chromatographically. But a specific analytical method has been designed to isolate the two compounds [47]. It is not infrequent to find pairs of coeluting isobaric PPAPs that are indistinguishable by conventional chromatography/mass spectrometry methods. Robust methods are often required to purify those isomers [48].

## Anticancer Properties of Oblongifolin C and Guttiferone K

Different pharmacological properties have been reported with oblongifolins and guttiferones, including modest antiviral effects observed with Ob-J and Ob-M [22] and antibacterial effects reported with different guttiferone derivatives, including Gt-A [19, 49]. This latter compound exerts also antifungal [50, 51] and antiparasite activities [30, 52, 53]. Gt-K has shown antioxidative properties in vitro, with a marked capacity to reduce peroxynitrite-induced lipid peroxidation in blood platelets [32]. In addition, the compound has revealed an anti-inflammatory activity, potentially useful to combat tuberculosis [54]. But the effects most often described and pronounced are anticancer effects, reported with both Ob-C and Gt-K. Here, we will focus on the anticancer properties of these two related compounds.

### Inhibition of Cancer Cell Proliferation and Tumor Growth

Ob-C and Gt-K exhibit anti-proliferative and pro-apoptotic properties against different cancer cell lines. Ob-C is a little more potent than Gt-K. In cervical cancer HeLa cells, the two compounds showed apoptosis-inducing effects at 20 μg/mL, but at 10 μg/mL only Ob-C remained active [23]. In this cellular system, the pro-apoptotic capacity of Ob-C is superior to that of the other oblongifolins, such as Ob-E/J [10]. Ob-C is more potent than Ob-A (Fig. 2) and Gt-K, and rapidly drives HeLa cells toward apoptosis via activation of a mitochondria-dependent pathway with activation of different caspases, such as caspases-3 and -8 [55]. Ob-C was found to induce the translocation of protein Bax, the release of cytochrome C and mitochondrial fission, with the characteristic activation of the intrinsic apoptotic pathway in HeLa cells [55]. In vitro, the antiproliferative action of Ob-C is less pronounced compared to that of established cytotoxic anticancer drugs like etoposide or paclitaxel, but interestingly, Ob-C was found to maintain a robust activity in cancer cells with a multi-drug-resistant (MDR) phenotype. For example, IC_50_ of 123.9 and 9.8 μM were determined with paclitaxel and Ob-C, respectively, with P-glycoprotein-overexpressing HCT-15 colon cancer cells in vitro [55]. Moreover, in a xenograft model of melanoma, Ob-C (60 mg/kg) was found to be equally potent to etoposide at reducing the growth of MDA-MB-435 tumor cells in mice, without inducing excessive weight loss [55].

The anticancer properties of Ob-C have been demonstrated using diverse tumor cell lines, notably with human cholangiocarcinoma QBC939 cells [56], colon carcinoma HCT116 cells [57], human cervical carcinoma HeLa cells [55] and various pancreatic cancer cell lines [58]. In each case, the natural product reduced cell proliferation in a dose-dependent manner and triggered mitochondria- and caspase-dependent apoptosis. The activity against pancreatic cancer cells was remarkable because the compound significantly reduced the growth of multiple cell lines with a good efficacy (IC_50_ of about 5.7, 7.8, 12.2, 8.0, and 7.0 μM after 48 h with MIA PaCa-2, Capan-1, SW1990, PANC-1, BxPC-3, respectively) but it also inhibited the growth of pancreatic cell lines resistant to gemcitabine. Ob-C markedly reduced the growth of gemcitabine-resistant MIA PaCa-2 tumors in mice, coupled with a downregulation of the Src protein [58]. Similarly, Gt-K reduced the growth of HT29, HCT116 and Colon-26 colon cancer cells with a roughly similar efficacy (IC_50_ of about 3 μM after 48 h) and triggered G_0_/G_1_ cell cycle arrest and apoptosis [59]. The compound alone revealed a modest antitumor activity in vivo, in a xenograft model of colon cancer (Colon-26, 10 mg/kg) but the activity was largely amplified when Gt-K was combined with 5-fluorouracil [59]. With no doubt, both Ob-C and Gt-K display a solid antitumor potential in murine experimental models.

### Regulation of Autophagy

Ob-C functions as a regulator of autophagy, more potently than Gt-K. In HeLa cells, Ob-C dose-dependently enhanced the number of autophagosomes and blocked their fusion with lysosomes, by altering lysosomal acidification and downregulating the expression of lysosomal cathepsins, thereby blocking the lysosomal proteolytic activity [60]. The natural product was found to function as an autophagic flux inhibitor capable of reducing the growth of cervical tumor in mice. In this case, the in vivo activity of Ob-C was relatively modest (about 40% tumor growth inhibition at 30 mg/kg) but the activity was markedly enhanced upon combination of Ob-C with a calory restriction scheme, to reach about 80% growth inhibition [60]. The regulation of autophagy seems to be a hallmark of Ob-C activity in cancer. Recently, Ob-C was found to enhance the anticancer activity of gemcitabine in RT-112-Gr bladder cancer cells initially resistant to this nucleoside analogue, by inhibiting autophagic flux. Ob-C reversed gemcitabine-resistance, and the effect was linked to the drug-induced inhibition of autophagy flux [61]. Ob-C might be useful to restore the sensitivity of chemo-resistant cancer cells to various cytotoxic drugs.

**Fig. 4:**
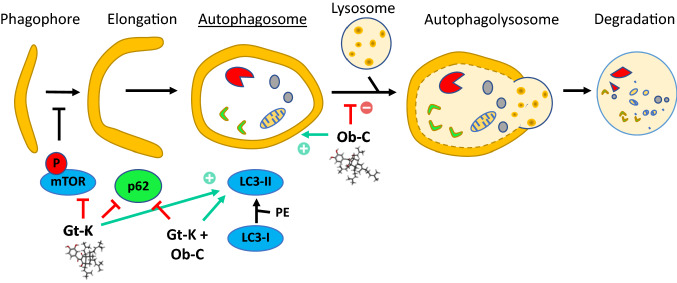
Effects of Ob-C and Gt-K on the autophagic flux. The two compounds induce the accumulation of autophagosomes, either by promoting their formation via degradation of p62 and inhibition of phospho-mTOR (Gt-K), or by blocking the fusion of autophagosomes with lysosomes (Ob-C). The combination of Ob-C and Gt-K strongly promote autophagy (by decreasing p62 and promoting LC3-II proteins)

Autophagy also plays an essential role in regulating Gt-K-mediated cell death. In HeLa cells, Gt-K induced characteristic signs of autophagy, with a marked induction of LC3 puncta formation, accumulation of autophagosomes-associated protein LC3-II and the degradation of the ubiquitin receptor p62 (also named sequestosome-1, SQSTM1) [62]. This compound is an autophagy inducer, not a suppressor like Ob-C [60, 62]. But both compounds trigger autophagy-dependent cell death. Gt-K has been found to inhibit Akt phosphorylation in HeLa cells and to induce the accumulation of reactive oxygen species. The compound blocks the mTOR (mammalian target of rapamycin) pathway in cancer cells [62]. The pro-autophagy activity of Gt-K has been evidenced also using cultured RAW264.7 macrophages. by inhibiting phosphorylation of proteins Akt and mTOR [54]. Therefore, despite their close structural similarity, the two PAPPs exert different effects on autophagy. However, in both cases the compounds trigger accumulation of autophagosomes, either because their formation is enhanced (Gt-K) or because their fusion with lysosomes is blocked (Ob-C) (Fig. 4).

The related PPAPs garcinol (Fig. 2), which is structurally close to Gt-K with a prenyl side chain at C-6 functions as an inhibitor of autophagy in human prostate cancer cells (like Ob-C) but its action is again different. It functions through activating p-mTOR and the p-PI3 Kinase/AKT pathway [63]. Another study indicated that garcinol can increase the autophagic flux through deacetylation of cellular proteins (inhibition of acetyltranferases) and inhibition of the mTORC1 pathway [64]. Therefore, garcinol appears structurally similar to Gt-K but functionally closer to Ob-C, at least in terms of autophagy modulation. Very recently, garcinol has been shown to function as a selective inhibitor of histone deacetylase 11 (HDAC11) [65] but apparently it can also affect other targets, such as topoisomerase II [66], monoamine oxidase-B [67] and the TLR4/NFĸB signal pathway [68]. Garcinol and isogarcinol display a range of biological effects [69–71].

A combination of Ob-C and Gt-K was found to induce both apoptosis and autophagy of HCT116 colon cancer cells [33]. The pair of compounds induced cleavage of PARP, a signature of caspase-activated apoptosis, and they caused an increase in LC3-II protein and a decrease in p62 protein in nutrient-deprived medium conditions (Fig. 4). Moreover, their pro-apoptotic effect was reduced in the presence of hydroxychloroquine, a prototypical autophagy inhibitor. The compounds combination showed an additive effect on the growth of HCT116 in vitro, and a marked capacity to reduce colony formation [33].

### Anti-Metastatic Activity

The initial study reporting the identification of Ob-A/D from *G. oblongifolia* indicated that the compounds inhibit tubulin assembly in vitro with a modest efficacy (IC_50_ in the 50–100 μM range). Ob-C was the most active compound of the four molecules, with an IC_50_ of 53 μM and it showed no effect on microtubule disassembly, unlike the taxane derivative paclitaxel [20]. The tubulin effect of Ob-C is weak in vitro but may play a role in the antimetastatic activity of the compound. Ob-C can inhibit metastasis in human esophageal squamous carcinoma Eca109 cells via tubulin aggregation. The compound increases the expression of the intermediate filament protein keratin-18 which then leads to an enhanced expression of its downstream effector tubulin [72]. Keratin 18 is believed to play an active role in cancer progression [73]. Ob-C dose-dependently reduces the invasion and migration capacity of Eca109 cells in vitro, and these drug effects are essentially abolished when cells are treated with a siRNA against keratin-18. In this cellular system, Ob-C was found to exert prominent antimetastatic effects, via an upregulation of keratin-18 and inhibition of phospho-MEK and phospho-AKT in vivo [72]. This type of effect is not restricted to Ob-C because a similar inhibition of cell migration has been reported with Ob-L (and the related compounds oblongixanthones D and E) associated with a down-regulation of phospho-MEK and phospho-ERK in human TE1 esophageal carcinoma cells [74]. Gt-K has also been found to exert anti-metastatic effects in hepatocellular carcinoma, through an up-regulation of the actin-binding protein profilin 1 (PFN1). The drug-induced restoration of aberrantly reduced PFN1 protein expression in an experimental model of HCC suppresses motility and metastasis of HCC cells [75].

## Molecular Targets of Ob-C and Gt-K

**Fig. 5:**
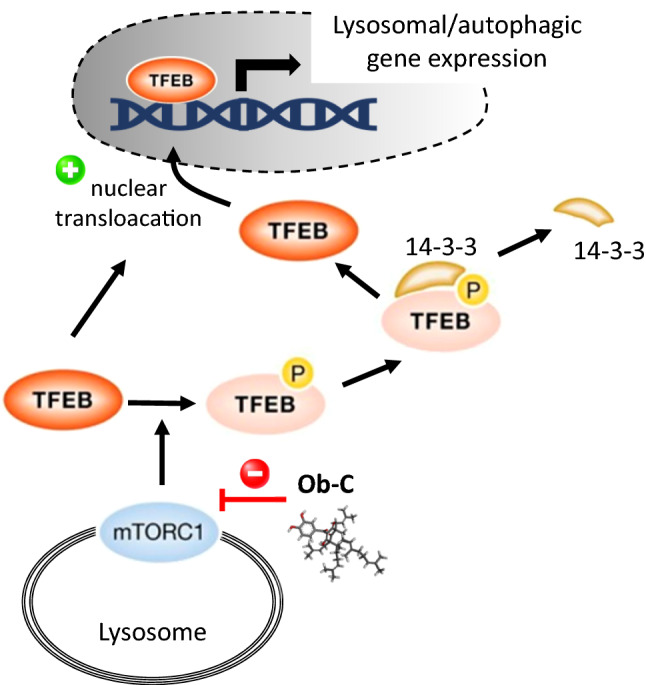
Ob-C induced nuclear translocation of the transcription factor EB (TFEB). The suppression of the expression of mTORC1 by Ob-C leads to the dissociation of TFEB/14–3-3 complex, thus the nuclear translocation of TFEB and then the expression of genes implicated in the autophagy process

The mechanism at the origin of the capacity of Ob-C (and Gt-K) to alter the lysosome-autophagy pathway is not precisely known at present but important information have been brought recently. The transcription factor EB (TFEB) seems to be chiefly implicated in this effect. TFEB can bind to the promoter of autophagy-associated genes and induces the formation of autophagosomes, autophagosome-lysosome fusion, and lysosomal cargo degradation. It is a master regulator of autophagy and TFEB agonists are actively searched to ameliorate diseases with autophagy dysfunction [76]. A variety of natural products, including Ob-C, have been found to function as TFEB activators capable of regulating the dysfunctions of the autophagy-lysosome pathway [77]. Specifically, Ob-C was found to enhance nuclear translocation of TFEB in HeLa cells and to reduce its interaction with 14–3-3 proteins [78]. It is likely the drug-induced suppression of mTORC1 (mammalian target of rapamycin complex 1) which favorizes the dissociation of TFEB/14–3-3 complex, thereby allowing the nuclear translocation of the transcription factor (Fig. 5). mTORC1 is an important regulator of the autophagy process, largely implicated in autophagosome formation. It also phosphorylates different transcription factors, including TFEB, thereby modulating their subcellular localization and transcriptional activities [79].

The mTOR cell survival pathway plays a central role in the biological activities of different PPAPs. Notably Gt-E and Gt-H were shown to inhibit the growth of human colon cancer cells (HCT116, HT29, SW480) by activating the endoplasmic reticulum stress response and inhibiting the mTOR cell survival pathway [80]. Another PPAP called garcimultiflorone K, from *Garcinia multiflora*, was found also to alter the Akt/mTOR signaling cascade implicated in its anti-angiogenic effect [81]. Moreover, recently the PPAP derivative nujiangefolin D was found to reduce the expression of phospho-mTOR in HeLa cells and a molecular modeling analysis suggested that the compound could directly interact with the mTOR serine/threonine-protein kinase [82]. There are also polyprenylated xanthone/benzophenone derivatives, such as gambogic acid and isogambogenic acid, known to trigger autophagy and/or apoptosis in cancer cells via the mTOR pathway [83, 84, 85]. The protein kinase mTOR, a central regulator of cell growth, may represent a direct target for the studied compounds, but for the time being, there is no experimental proof of a potential molecular interaction between mTOR and Ob-C or Gt-K.

On the opposite, there are robust experimental evidence that Ob-C can bind to the heat shock 70 kDa protein family A member 8 (HSPA8, also known as Hsc70) and to a much lower extent to the cysteine-protease cathepsin B. These two protein targets were identified in the frame of a protein-fishing study with Ob-C and fully characterized as Ob-C interacting proteins. Ob-C potently interacts with the purified HSPA8 protein in vitro (Kd = 6.2 μM and 11.8 μM, measured by ITC and SPR, respectively) and can also bind purified cathepsin B (Kd = 25.6 μM and 39.3 μM by ITC (isothermal titration calorimetry) and SPR (surface plasmon resonance), respectively) [86]. Under heat shock stress, Ob-C inhibited the nuclear translocation of HSPA8 and significantly increased the expression level of the tumor suppressor p53 in A549 cancer cells. Moreover, the compound promoted the interaction between p53 and HSPA8, and significantly enhanced apoptosis in cisplatin-treated cells [86]. This key study strongly supports the idea that HSPA8 is a primary target of Ob-C.

**Fig. 6:**
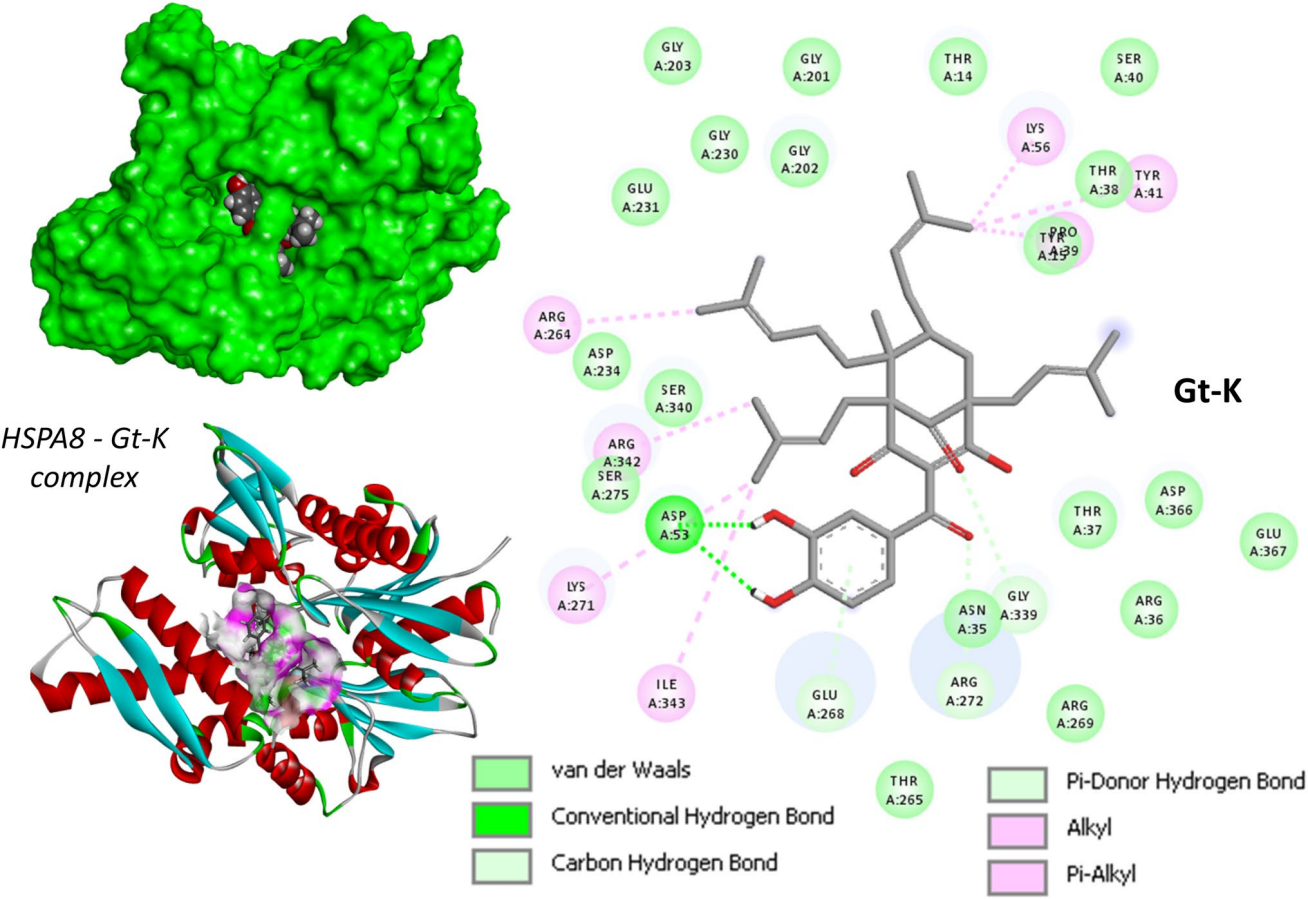
Molecular model for the binding of Gt-K to HSPA8. The structure derives from the crystal structure of the ATPase fragment of a 70K heat-shock cognate protein solved by X-ray diffraction (PDB code: 3HSC) [86]. A binding site centered on residue Arg272 was identified using the software Discovery Studio Visualizer, to map the position of the cavities susceptible to accommodate the ligand. Within the binding site, side chains of specific amino acids have been considered as fully flexible. The flexible amino acids are Tyr15, Lys71, Glu231, Phe233, Glu268, Lys271, Arg272, Phe302, Arg342 and Asp366. The protein surface model is shown in green with the drug bound (top left). A model of the hydrogen-bond donor pink) and acceptor (green) surfaces is presented (bottom left). The binding map contact for Gt-K bound to HSPA8 is also shown with the indicated color code (right). The modeling analysis was performed as recently described [87, 88]

**Fig. 7:**
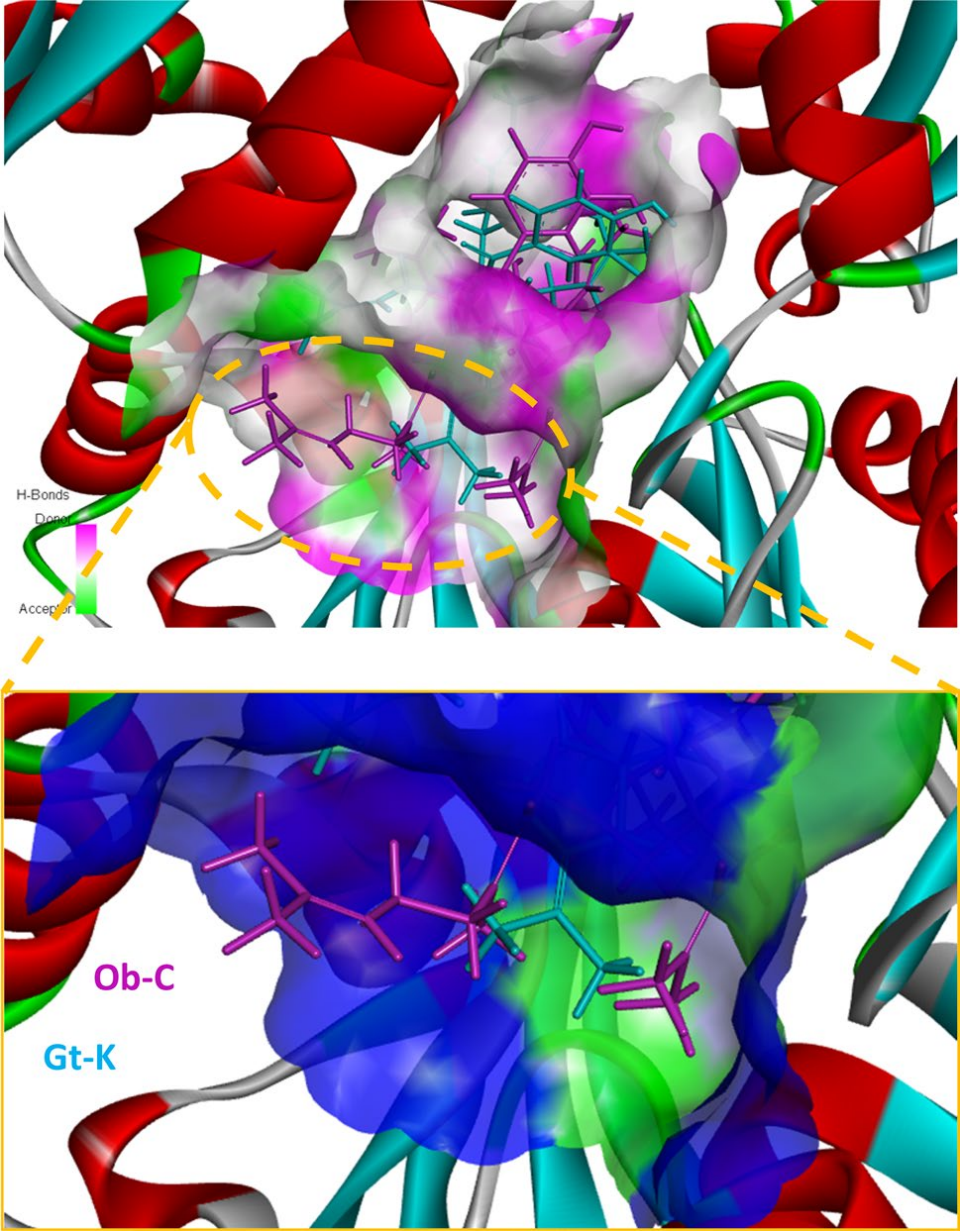
Molecular model of Gt-K and Ob-C within the binding site of HSPA8. The top view shows the two compounds superimposed, with the hydrogen-bond donor (pink) and acceptor (green) surfaces. The bottom view is a focus on the C-6 prenyl (Gt-K) and geranyl (Ob-C) side chain which tends to point toward the exterior of the drug-binding cavity

We have performed a molecular modeling analysis of the binding of both Ob-C and Gt-K to the N-terminal nucleotide binding domain (NBD) of HSPA8. The structure of this protein domain (ATPase fragment) has been solved by X-ray diffraction (PDB code: 3HSC) [87], offering a frame to dock potential binder. A molecular model of Gt-K bound to HSPA8 NBD is presented in Fig. 6 (modeling analysis performed as previously described [88, 89]). A similar model was obtained with Ob-C (not shown). Both compounds can form stable complexes with the protein, but we found that the protein complexes formed with Gt-K were more stable than those observed with Ob-C. The calculated empirical energies of interaction (ΔE) were − 98.1 kcal/mol for Gt-K versus − 86.7 kcal/mol for Ob-C bound to HSPA8 (and the free energy of hydration (ΔG) is almost identical, ΔG = − 23.2/− 24.3 kcal/mol. We also modeled the keto-enol tautomerism of the C-10 carbonyl function, comparing the binding to HSPA8 of the two enol tautomers. But here again, we observed that Gt-K (enol) could form more stable complexes with HSPA8 than Ob-C (enol). The calculated empirical energies of interaction (ΔE) were − 104.4 kcal/mol for Gt-K (enol) versus − 96.3 kcal/mol for Ob-C (enol) bound to HSPA8. The two compounds Ob-C and Gt-K bind to a central hydrophobic pocket of HSPA8. Their respective positions within the binding site are slightly distinct but, in both cases, we could identify more than 25 potential drug-protein interactions, including conventional H-bonds, van der Waals contacts, and alkyl interactions (Fig. 6). The two compounds occupy the same hydrophobic cleft in the protein. The Gt-K molecule is almost completely buried into the groove delimited by two α-helices, whereas the longer geranyl side chain of Ob-C exits the groove to point toward the exterior of the protein surface, as represented in Fig. 7.

As a molecular chaperone, HSPA8 has a variety of cellular functions. The protein is not only involved in autophagy but also in protein quality control, protein import and immunity [90]. The protein has now emerged as a valuable pharmacological target in a number of autoimmune situations. Various activators and inhibitors of HSPA8 have been identified. Many of the inhibitors target the NBD domain of HSPA8, and at least two of them have been evaluated in patients: the spergualin derivative 15-DSG which is a potent immunosuppressant, and the 21-mer phosphopeptide P140 (lupuzor), both tested in patients suffering from systemic lupus erythematosus [91]. The concept is to remodel the autophagy-lysosomal pathway which is altered in lupus and other (auto)inflammatory diseases [92, 93]. A compound like Ob-C (and likely Gt-K) which targets HSPA8, could thus be useful to regulate immunity, in addition to enhancing the effects of anticancer drugs. For example, the related compound Gt-J has been found to trigger a strong immunomodulation, inducing a marked decrease of major histocompatibility complex (MHC class II) molecules on endothelial cells [94] and the related compound Gt-F has revealed also immunoregulatory activities, downregulating the expression of several MHC molecules at the surface of human primary endothelial cells upon inflammation [6]. Altogether, this information should encourage further study of the immunoregulatory effects of Ob-C and its capacity to interact with HSPA8. However, HSPA8 is probably not the only potential target for Ob-C and Gt-K. These two compounds exhibit a range of bioactivities, including anti-inflammatory effects for example [54, 95]. There are probably different proteins implicated in their pharmacological effects, as it is frequently the case with natural products.

## Conclusion

Ob-C and Gt-K are the two main components of the ethanol extract from the Chinese plant *Garcinia yunnanensis*. This plant, used in traditional Chinese medicine, possesses potent anti-inflammatory and anticancer activities by regulating multiple signaling pathways [95, 96]. These two compounds are among the most potent anticancer bicyclic polyprenylated acylphloroglucinols (BPAPs) reported thus far. A few other compounds, such as Ob-L, Gt-E and Gt-H also revealed also antiproliferative activities, but they are less potent than Ob-C and Gt-K [97]. The potency of these compounds is coherent with the long-established use of the plant *Garcinia oblongifolia* Champ. ex Benth (which contains both oblongifolins and guttiferones) in traditional Chinese medicine [11]. *G. oblongifolia* extracts represent a rich source of PPAPs. A single extract can contain up to 120 PPAPs, with Ob-C and Gt-K being the most abundant [48, 73]. These two compounds can be isolated, and their mechanism of action studied, taking advantage of robust methods such as the use of molecularly imprinted polymers [45].

Ob-C was initially identified from a sample of *G. oblongifolia* collected in Vietnam and the first biochemical effect evidenced was a mild inhibition of tubulin assembly, in vitro [20]. For Gt-K, initially isolated from a plant of the Madagascar rain forest (*Rheedia calcicola* Jum. & H. Perrier), an antiproliferative activity against cancer cells was initially reported but no target was proposed at that time [34]. Fifteen years have passed since their first isolation and we have now acquired a much better knowledge of their complex mechanism of action, implicating several targets and pathways. It is clear that the regulation of autophagy is central to the compounds’ mechanism of action. They both induce an accumulation of autophagosomes in cells, apparently via a distinct action: inhibition of autophagosome/lysosome fusion for Ob-C, induction of autophagosome formation for Gt-K.

An interesting parallel between the two acylphloroglucinol derivatives discussed here and the drug phloroglucinol can be underlined. Phloroglucinol (1,3,5 tri-hydroxy-benzene) is a musculotropic anti-spasmodic drug, prescribed (in the European Union) for alleviating abdominal pain [98]. Phloroglucinol can induce the formation of autophagosomes in follicle cells [99] and an anticancer phloroglucinol derivative (PMT7) was found to kill glycolytic cancer cells by blocking autophagy [100]. Moreover, the apple dihydrochalcone phloretin, which possesses a phloroglucinol moiety linked to phenol side chain, is also an autophagy-inducer active against drug-resistant cancer cell lines [101], as observed with Ob-C [58]. The phloroglucinol moiety of Ob-C and Gt-K could well be the “autophagy regulatory unit” of the compounds.

The most innovative part of the mechanism of action of Ob-C and Gt-K is linked to the heat shock protein HSPA8. Ob-C binds tightly to this chaperone protein [86] and our modeling analysis suggests that Gt-K could be an even better HSPA8 binder than Ob-C. On the one hand, this information brings new ideas for the potential use of the natural products in different pathologies, taking advantage of the multiple functions of HSPA8 in cancer and in autoimmune diseases, as mentioned above [90]. HSPA8 is also a host factor involved in infectious bronchitis virus (IBV, a coronavirus) infection [102]. HSPA8 is a regulator of the life cycle of the enterovirus A71 (EV-A71) [103] and Ob-M can potently inhibit the reproduction of this virus [104]. But on the other hand, the binding of Ob-C to HSPA8 also raises questions because HSPA8 is an accessory protein expressed in many tissues, including pulmonary vein cardiomyocytes [105]. This heat shock protein plays a role in cardiac function after ischemia–reperfusion [106]. Interfering with its multiple functions could thus be a challenge. Nevertheless, Ob-C and Gt-K are interesting natural products, endowed with potent anticancer properties. They deserve further studies.

## Abbreviations

*Gt-K*: Guttiferone K
*HSPA8*: Heat shock protein family A member 8
*Ob-C*: Oblongifolin C
*PPAPs*: Polycyclic polyprenylated acylphloroglucinols
